# Association of Mandible Anatomy with Age, Gender, and Dental Status: A Radiographic Study

**DOI:** 10.5402/2013/453763

**Published:** 2013-12-18

**Authors:** Revant H. Chole, Ranjitkumar N. Patil, Swati Balsaraf Chole, Shailesh Gondivkar, Amol R. Gadbail, Monal B. Yuwanati

**Affiliations:** ^1^Department of Oral Medicine and Radiology, Kalinga Institute of Dental Sciences, Bhubaneshwar, Odisha 751024, India; ^2^Department of Oral Medicine and Radiology, King George Medical College, Lucknow, Uttar Pradesh 226003, India; ^3^Department of Public Health Dentistry, Shri Aurobindo Institute of Dental Sciences, Indore, Madhya Pradesh 453111, India; ^4^Department of Oral Medicine and Radiology, MGVM's Dental College, Nashik, Maharashtra 422003, India; ^5^Department of Oral Pathology and Microbiology, Sharad Pawar Dental College, Wardha, Maharashtra 442004, India; ^6^Department of Oral Pathology, Peoples Dental Academy, Bhanpur Bypass Road, Bhopal, Madhya Pradesh 462037, India

## Abstract

*Introduction*. Gonial angle and antegonial region are important landmarks in mandible which is influenced by gender, age, and dental status. The objective of this study was to evaluate the gonial angle, antegonial angle, and antegonial depth and to investigate their relationship to gender, age group, and dental status. *Materials and Methods*. A total of 1060 panoramic radiographs were evaluated: the dentulous group, 854 subjects and the edentulous group, 206 subjects. The patients were grouped into six age groups of 10-years each. Gonial angle, antegonial angle, and antegonial depth were measured from panoramic radiographs. *Results and Discussion*. Corelation of age with gonial angle, antegonial angle and antegonial depth was not significant. Significant difference in mandibular angle was found between males and females. Males had significantly smaller antegonial angle and greater antegonial depth than females. Significant difference was found for gonial angle, antegonial angle, and antegonial depth between right and left sides of mandible. *Conclusion*. Gonial angle, antegonial angle, and antegonial depth can be implicated as a forensic tool for gender determination but not suitable for age determination.

## 1. Introduction

Various authors have described number of changes that take place in the morphology of the human mandible with advancing age. One of the prominent changes that have been suggested is the change in the gonial (mandibular) angle. The angle between the ramus and the corpus of the mandible is called the gonial angle. A surface field of resorption is present on the inferior edge of the mandible at the ramus-body junction, forming the antegonial notch. Any change in the gonial angle is largely produced by ramus remodeling and is determined by the remodeling direction of the ramus with its condyle. Very few studies have been carried out to correlate the changes in the mandibular angle with age, sex and dental status [1–5]. Previous reports on widening of the gonial angle in edentulous patients are conflicting. Aside from age and loss of teeth, other factors may influence change in gonial angle. Panoramic radiograph is the most obvious choice for determination of the gonial angle [4]. Thus, the purpose of this study was to evaluate gonial angle, antegonial angle, and antegonial depth from panoramic radiographs of normal subjects and to investigate their relationship to gender, age group, and dental status.

## 2. Materials and Methods

This study evaluated 1060 panoramic radiographs of patients visiting Sharad Pawar Dental College and Hospital, Wardha, India, and RKDF Dental College and Research Centre, Bhopal, India. Intraoral examination of the subjects was done to rule out attrition, traumatic bite, malocclusion, bruxism, or any temporomandibular joint anomaly. Patients with fractures, postsurgical cases, and with any acquired skeletal deformities were excluded from the study. The subjects were grouped into six age groups of 10 years each. The youngest age group was 15–25 years and the oldest was 66 years and above (Table 1). All the panoramic images were made using the Planmeca Proline CC Panoramic X-ray, Helsinki, Finland. Gonial angle, antegonial angle, and antegonial depth were measured on panoramic radiographs by a single dentomaxillofacial radiologist.

**Figure 1:**
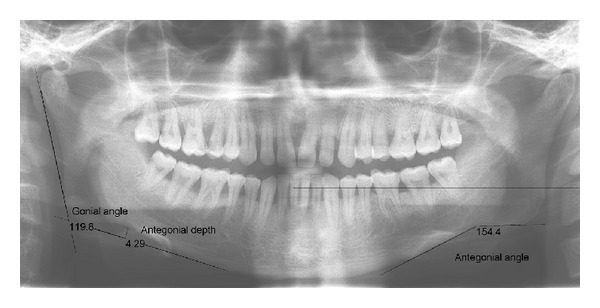


The mandibular line was constructed as a tangent to the two lowest points on the anterior and posterior borders of the mandible. The ramus line was constructed through the two most distal points of the ramus. The intersection of these lines formed the gonial (mandibular) angle. The antegonial angle was measured by two lines parallel to the antegonial region that will intersect at the deepest point of the antegonial notch. The antegonial depth was measured as the distance along a perpendicular line from the deepest point of the notch concavity to a tangent through the inferior border of the mandible (Figure 1).

## 3. Results and Discussion

Statistical analysis was carried out by using student's unpaired *t*-test. Correlation of age with gonial angle, antegonial angle, and antegonial depth was not significant (*P* > 0.05). Significant difference in gonial angle was found between males and females. Gonial angle in males was found to be 118.056° ± 6.47 and in females was 123.109° ± 7.439 (*P* < 0.05). Males had significantly smaller antegonial angle than females (162.2° ± 7.39 and 167.52° ± 6.27, resp.) and significantly greater antegonial depth than females (2.251 mm ± 1.405 and 1.14 mm ± 0.5763, resp.), irrespective of the dental status (*P* < 0.05) (Table 2). Significant difference was found for gonial angle, antegonial angle, and antegonial depth between right and left sides of mandible (*P* < 0.05) (Table 3).

Previous literature suggests that, as age advances, the gonial angle decreases and becomes less obtuse in adulthood and again increases as the age advances towards old age. Cross-sectional studies have promoted the view that the gonial angle is increased by age and by the edentulous state. Longitudinal studies do not support this view [1]. In this study, in males, the gonial angle decreased up to 55 years and became obtuse as the age advances. In females, the gonial angle decreased up to 55 years, increased in 56–65 years, and again decreased above 65 years of age.

The literature shows discrepant results concerning the changes in the gonial angle with age and dental status. Casey and Emrich [2] found no statistical significant difference in gonial angle in the edentulous and dentulous sides. Their results suggested slight widening of the mandibular angle in the edentulous patients. Similar results were found by Ohm and Silness [3] who showed that the edentulous participants had the largest mean angle, as compared to the participants in possession of all teeth. The partially dentate participants had a jaw angle size between that of the aforementioned groups. Preliminary results of the analysis (ANOVA) showed that the number of teeth had a decisive influence on the size of the gonial angle. The correlation coefficients between size of the gonial angle and age showed that age explained approximately 8–16% of the variation of the angle through its relation with age. Sex differences in age and size of the gonial angle were not statistically significant in any of the three tooth retention categories. Xie and Ainamo [6] found difference in size of the gonial angle between dentate men and women (*P* < 0.05 in the young and *P* < 0.001 in the older dentate group) but not between elderly edentulous men and women. The elderly edentulous subjects had significantly larger gonial angles (128.4 degrees ± 6.6) than did the young (122.4 degrees ± 6.6, *P* < 0.001) and older dentate subjects (122.8 degrees ± 6.6, *P* < 0.001). These results were slightly contradictory to our results. Raustia and Salonen [7] measured the gonial angles of the mandible and condylar and ramus heights of 30 complete denture wearers (18 women, 12 men, mean age 61 years, range 42–74 years) coming for renewal of their dentures, using panoramic radiographs. No statistically significant difference was observed between the sexes in the sizes of gonial angles and condylar and ramus heights. Our results corelated with Huumonen et al. [8] who found significantly larger gonial angle in females as compared to males. However, in their study in edentulous subjects, the gonial angle was significantly larger, while the ramus and condylar heights were significantly smaller on both sides compared with dentate subjects. Ceylan et al. [9] found no significant differences between the mandibular angles when comparing partially edentulous and totally edentulous subjects. In our study, the gonial angle was associated with gender but not with age and dental status. Our results did not corelate with Baydaş et al. [10] who found no statistically significant gender differences in gonial angle and antegonial notch depth. Francis Fish [1] proposed that the gonial angle may show enlargement or reduction, as may be expected of any bony angular relationship, and that ageing and loss of teeth are not, and should not be expected to be, the sole determinants of such change. A recent study showed that gonial angle decreased significantly with age from 140.17° ± 5.9° (primary dentition) to 123.61° ± 6.9° (late permanent dentition; *P* < 0.001) [11]. Shahabi et al. [12] showed that the mean value of the gonial angle in the panoramic radiograph was 124.17° with a standard deviation of 5.87°. The gonial angle in males was 123.68° and that in females was 124.39° with no statistically significant difference between the two genders. The mean value of the right gonial angle was 123.94° with a standard deviation of 6.20° and the mean value of the left gonial angle was 124.40° with a standard deviation of 5.88°. However, there was no statistically significant difference between the right and left gonial angles (*P* = 0.670). This result did not corelate with our study in which there was a statistically significant difference between the right and left gonial angles (*P* < 0.05). Evaluation of the gonial angle in the Anatolian populations by Gungor et al. [13] showed that there were no significant differences between the right and left gonial angles of the individuals, but there was a significant difference at the left gonial angle between sexes (*P* < 0.01). Yanikoğlu and Yilmaz [14] showed that the gonial angle values tend to increase in both sides after tooth extractions, while it decreases after one year of tooth extraction.

Mattila et al. [4] demonstrated that the size of the gonial angle can be determined from the orthopantomogram with the same degree of accuracy as from the generally used lateral cephalogram. It also showed that the right and left gonial angles can be quite easily determined individually from orthopantomogram, thus avoiding the disturbing influence of the superimposed images found on lateral cephalograms.

The morphological change in the antegonial region has received little attention in the literature. According to Dutra et al. [5], for antegonial angle, the males (160.86° ± 0.78) had significantly smaller values than females (165.08° ± 0.58) irrespective of the dental status (*P* < 0.0001). Edentulous individuals (161.51° ± 0.83) had a smaller antegonial angle than dentate (165.05° ± 0.76) and partially dentate (163.81° ± 0.81) individuals (*P* < 0.05). In a recent study by Ghosh et al. [15] in both males and females, on right side as well as left side, there was no statistically significant change in the value of the antegonial angle with respect to age, although a trend of decrease in the antegonial angle with age was observed. The mean difference between the antegonial angle value at the age of 20–29 years and 60 years and above was 1.33° in males and 0.93° in females. Similarly, with respect to the antegonial depth, the mean value did not change significantly with age. Females had higher values of antegonial angles as compared to males. With respect to antegonial depths, females had smaller values as compared to males. According to Dutra et al. [5], the antegonial depth was significantly greater for males than females (2.12 mm ± 0.09 versus 1.46 mm ± 0.07, *P* < 0.0001). Edentulous individuals (1.87 mm ± 0.1) had significant greater antegonial depth than dentate and partially dentate individuals (1.60 mm ± 0.1 and 1.65 mm ± 0.1, resp.). Our results strongly support these findings. In our study, males had significantly smaller antegonial angle and greater antegonial depth than females.

This might be due to gender hormonal differences affecting bone metabolism.

## 4. Conclusion

This study showed that the gonial angle and antegonial region are influenced by gender but not by age and dental status. Thus, changes taking place in gonial angle, antegonial angle, and antegonial depth can be implicated as a forensic tool for gender determination but not suitable for age determination.

## Figures and Tables

**Table 1:** Sample distribution by gender, age group, and dental status.

Characteristics	Number of patients	Percentage (%)
Gender		
Male	566	53.39
Female	494	46.60
Age group (yrs.)		
15–25	240	22.64
26–35	188	17.73
36–45	174	16.41
46–55	130	12.26
56–65	162	15.28
66 and above	166	15.66
Dental status		
Dentulous	854	80.56
Edentulous	206	19.43

**Table 2:** Distribution by gender and dental status (mean and standard deviation) for gonial angle, ante gonial angle, and ante gonial depth (mm).

Characteristics	Mean	SD	*t* value	*P* value
Gonial angle				
Gender				
Male	118.056	6.47	3.58	Significant *P* < 0.05
Female	123.109	7.439		
Dental status				
Dentulous	120.53	7.38	1.147	Not significant *P* > 0.05
Edentulous	118.38	7.84		

Ante gonial angle				
Gender				
Male	162.2	7.39	3.848	Significant *P* < 0.05
Female	167.52	6.27		
Dental Status				
Dentulous	164.81	7.34	1.107	Not significant *P* > 0.05
Edentulous	163.11	6.02		

Ante gonial depth (mm)				
Gender				
Male	2.251	1.405	5.14	Significant *P* < 0.05
Female	1.14	0.5763		
Dental Status				
Dentulous	1.76	1.22	1.23	Not significant *P* > 0.05
Edentulous	2.04	0.84		

**Table 3:** Mean and confidence interval of the gonial angle, antegonial angle, and antegonial depth.

Characteristics	Right		Left		Total	
	Mean	SE	Mean	SE	Mean	SE
Gonial angle	119.97	0.742	121.08	0.88	120.5	0.745
*t* calculated value	7.22, significant, P* < 0.05					
Ante gonial angle	164.88	0.775	163.22	1.87	164.81	0.741
*t* calculated value	10.18, significant, P* < 0.05					
Ante gonial depth (mm)	1.79	0.129	1.23	0.124	1.73	0.123
*t* calculated value	9.108					
*t* tabulated value	1.98, significant, P* < 0.05					

*Comparison between right and left sides: *P* < 0.05 (significant).

SE: standard error of mean.
